# Are benefits and harms in mammography screening given equal attention in scientific articles? A cross-sectional study

**DOI:** 10.1186/1741-7015-5-12

**Published:** 2007-05-30

**Authors:** Karsten Juhl Jørgensen, Anders Klahn, Peter C Gøtzsche

**Affiliations:** 1 The Nordic Cochrane Centre, Department 3343, Rigshospitalet, Blegdamsvej 9, DK-2100 Copenhagen, Denmark

## Abstract

**Background:**

The CONSORT statement specifies the need for a balanced presentation of both benefits and harms of medical interventions in trial reports. However, invitations to screening and newspaper articles often emphasize benefits and downplay or omit harms, and it is known that scientific articles can be influenced by conflicts of interest. We wanted to determine if a similar imbalance occurs in scientific articles on mammography screening and if it is related to author affiliation.

**Methods:**

We searched PubMed in April 2005 for articles on mammography screening that mentioned a benefit or a harm and that were published in 2004 in English. Data extraction was performed by three independent investigators, two unblinded and one blinded for article contents, and author names and affiliation, as appropriate. The extracted data were compared and discrepancies resolved by two investigators in a combined analysis. We defined three groups of authors: (1) authors in specialties unrelated to mammography screening, (2) authors in screening-affiliated specialties (radiology or breast cancer surgery) who were not working with screening, or authors funded by cancer charities, and (3) authors (at least one) working directly with mammography screening programmes. We used a data extraction sheet with 17 items described as important benefits and harms in the 2002 WHO/IARC-report on breast cancer screening.

**Results:**

We identified 854 articles, and 143 were eligible for the study. Most were original research. Benefits were mentioned more often than harms (96% vs 62%, P < 0.001). Fifty-five (38%) articles mentioned only benefits, whereas seven (5%) mentioned only harms (P < 0.001). Overdiagnosis was mentioned in 35 articles (24%), but was more often downplayed or rejected in articles that had authors working with screening, (6/15; 40%) compared with authors affiliated by specialty or funding (1/6; 17%), or authors unrelated with screening (1/14; 7%) (P = 0.03). Benefits in terms of reduced breast cancer mortality were mentioned in 109 (76%) articles, and was more often provided as a relative risk reduction than an absolute risk reduction, where quantified (45 articles (31%) versus 6 articles (3%) (P < 0.001)).

**Conclusion:**

Scientific articles tend to emphasize the major benefits of mammography screening over its major harms. This imbalance is related to the authors' affiliation.

## Background

It is essential to consider both benefits and harms carefully when the merits of any intervention are discussed in the medical literature. This was recently emphasised in an extension to the CONSORT statement on reporting randomised trials [1], but it also applies to other scientific work, and to information material directed towards healthy citizens and patients [2]. However, the harms of breast cancer screening are often downplayed or left unmentioned in information materials, thus providing an inadequate basis for informed decision-making [3-7]. A similar bias has been found in newspaper articles on breast cancer screening [8]. This focus on benefits is contrary to the ethical imperative that those subjected to any preventive measure or treatment should receive unbiased information [2], and it is particularly troublesome in screening, as this is directed towards healthy individuals, not patients seeking treatment. The 2002 WHO/IARC report on breast cancer screening notes that "...the vast majority of women undergoing screening do not have breast cancer at the time of the examination, and these women cannot derive a direct health benefit from screening; they can only be harmed" [9].

This report also notes that "An obvious source of harm associated with any screening programme is unnecessary treatment of cancers that were not destined to cause symptoms" [9] and that "Overdiagnosis refers to the detection of cancers that would never have been found were it not for the screening test. Patients in whom such indolent cancers are detected do not benefit from screening and can only experience harm: the worry associated with a cancer diagnosis and the complications of therapy" [9]. Overtreatment is the treatment of such lesions. Overdiagnosis is different from identification of false positive cases, which refers to women recalled for further testing who do not have a suspicion of cancer confirmed. We were particularly interested in the issue of overdiagnosis as it markedly influences the life of women who experience it, could affect a substantial number of women [10], and because it is not mentioned in invitations to screening [7].

We were also interested in the potential conflicts of interest, including the medical specialties of the authors [11,12]. A relationship between conclusions and conflicts of interest has been demonstrated for passive smoking [13], anti-inflammatory drugs in rheumatoid arthritis [14], spinal manipulation [15], interventions to reduce allergens from house dust mites [16] and cardiovascular trials [17]. Authors also prefer references that support their preconceived opinions [18,19].

We examined articles in medical journals discussing mammography screening to see if they gave equal attention to benefits and harms, as we suspected that information materials and newspaper articles would reflect their presentation of the issues. We also explored the affiliation and source of funding of the authors to examine if they were associated with the likelihood of mentioning, or the framing of, benefits and harms. We experienced some challenges that led to supplementary analyses, which we also report.

## Methods

We included all articles on mammography screening that described a benefit or a harm, or both, that were published in English in 2004 and were identified in a comprehensive PubMed search; (exp breast neoplasm/all OR "breast cancer" OR exp mammography/all OR mammograph*) AND (exp mass screening/all OR screen*). We excluded articles on mammographic techniques, assessments of methodological approaches to evaluate screening, letters, and articles written by ourselves. The PubMed search was performed in April 2005 and was limited to articles published in 2004 to obtain a manageable sample size that was as up to date as possible. Two investigators read titles and abstracts independently. Potentially eligible articles were collected in hardcopy and read in full.

Data extraction was performed in parallel and independently by the three authors. One author (PG) was blinded to author affiliation when evaluating contents, and blinded to contents when evaluating author affiliation. The other two authors (KJJ and AK) were not blinded. Blinding was obtained by cutting out all information from the article on author names, affiliation and funding, and presenting this information separately from the contents. The blinded author noted all instances where he thought he could guess who the authors were or to which conflict of interest category they belonged (see below). Extracted data from the two unblinded authors were compared and consensus reached where there was disagreement ("unblinded consensus"). Subsequently, unblinded consensus data and data from the blinded extraction were compared and conflicts resolved by two of the authors (KJJ and PG) using the blinded material for reference. We analyzed our data in three ways: (1) data extracted by the blinded author, (2) data extracted by unblinded authors, (3) data combined from all three authors. This analysis will be referred to as the "combined analysis".

We extracted data on 17 items described as important benefits and harms in the 2002 WHO/IARC-report on breast cancer screening and used their definitions [9] (Table 1). For our primary analysis addressing whether potential benefits and harms of mammography screening were mentioned, we classified articles as: (1) only benefits mentioned, (2) only harms mentioned, (3) both benefits and harms mentioned. Mentioning one benefit or one harm was considered sufficient for our labelling. We also classified the framing of the discussion of harms as "mentioning harms", "acknowledging harms", or as "downplaying harms". An article mentioning one or more harms, in any way, would be labelled as "mentioning harms". Some of these were also labelled as "downplaying harms", because they used framing that made the harm seem unimportant, e.g. by describing it as negligible or non-existent (see Additional file 1 for examples). Those who did not downplay harms by framing were considered to acknowledge them.

**Table 1:** Benefits and harms of mammography screening included in our data extraction sheet and mentioned as important in the 2002 WHO/IARC report on mammography screening [9]

**Benefits**
Breast cancer mortality reduction
Relative risk reduction
Absolute risk reduction
Survival time from diagnosis
Number needed to screen
Carcinoma in situ, as positive
Less mastectomies/more tumourectomies
Total mortality reduction
Other


**Harms**
Overdiagnosis and overtreatment
Recall rate:
1. Per screen
2. Cumulative
More mastectomies/more tumourectomies
Increased biopsy rate
Risks associated with additional radiotherapy
Psychological effects related to false positives
Carcinoma in situ, as negative
Pain at mammography
Other

We classified articles in three author affiliation categories: (1) articles where all authors were in specialties unrelated to mammography screening, (2) authors in screening-affiliated specialties (radiology or breast cancer surgery) who were not working with screening, or authors funded by cancer charities, or (3) articles where at least one author worked directly with mammography screening. In many cases, author affiliation was not clear from the articles, or it was not clear if the institution employing the author performed screening. We therefore retrieved additional information from the Internet for our combined analysis. We were not able to find this for all authors, however, and these cases were labelled as not being affiliated with screening.

During our study, we realised that a blinded data extraction would be necessary to guard against bias pertaining to knowledge of author names or affiliation while evaluating if a particular harm was downplayed or acknowledged. Conversely, knowing the contents of an article could potentially influence our evaluation of author affiliation. In a few cases, we knew the author had previously worked with screening. If such articles were sceptical towards screening, we could be prone to label the author affiliation according to current status, and conversely if the article was supportive of screening. We therefore took care not to let our knowledge of the authors influence our judgments, and used the authors' current affiliation in all cases.

We used a chi-square test for trend [20] to compare the three conflict of interest groups for potential differences in the emphasis on benefits and harms, and a sign test to compare mutually exclusive events in the same group that would be expected to be similarly distributed in the absence of a preference for either when exploring potential differences among author affiliation groups in the number of times the separate 17 items were mentioned and acknowledged.

## Results

Our PubMed search yielded 854 titles and abstracts, 159 of which were considered eligible for further review and collected in hardcopy for assessment. Sixteen were subsequently discarded, 13 because they were letters and three because the affiliation of the author could not be established. Hence, 143 articles were evaluated. Original research articles predominated, and there was only minor variation between author groups regarding the type of article (original research, editorial, etc.; Table 2). Unless stated otherwise, all results relate to analyses using the combined data for all three authors.

**Table 2:** Distribution of article types, according to author affiliation (blinded consensus)

	Work unrelated to screening		Screening-related specialty		Working with screening	
	No. of articles	(%)	No. of articles	(%)	No. of articles	(%)

Review	8	(17)	1	(4)	9	(13)
Editorial	5	(10)	2	(7)	5	(7)
Original research	30	(63)	22	(81)	53	(78)
Other (e.g. commentaries)	5	(10)	2	(7)	1	(1)
Total	48	(100)	27	(99*)	68	(99*)

*Rounding error: should be 100% of articles.

Most authors preferred to focus on the benefits of mammography screening, which were mentioned more often than harms (96% vs. 62%, P < 0.001; Table 3). Fifty-five articles (38%) mentioned only benefits, whereas 7 (5%) mentioned only harms (P < 0.001). The benefit mentioned most often was a reduction in breast cancer mortality (109 articles, 76%). This benefit was more commonly presented as a relative risk reduction than as an absolute risk reduction, where quantified (45 articles, 31%, versus 6 articles, 3%)(P < 0.001). Five articles mentioned both the absolute and relative risk reduction, and one the absolute risk reduction only.

**Table 3:** Number and proportion of the 143 articles eligible for review that mention benefits, mention harms, and acknowledge harms of screening

	Unblinded analysis		Blinded analysis		Combined analysis	
	No. of articles	(%)	No. of articles	(%)	No. of articles	(%)

Mention benefits	139	(97)	134	(94)	137	(96)
Mention harms	99	(69)	84	(59)	89	(62)
Acknowledge harms	63	(44)	41	(29)	56	(39)
Total	143	(100)	143	(100)	143	(100)

**Figure 1:**
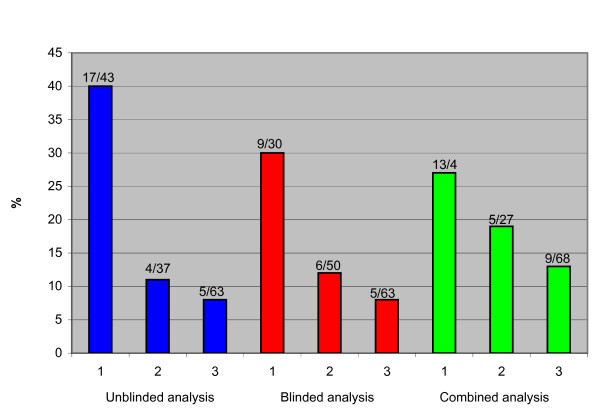
Number and proportion of the 143 articles eligible for review that acknowledge overdiagnosis, related to author affiliation. 1: Authors with no apparent conflict of interest. 2: Authors in screening-affiliated specialty or funded by cancer charities. 3: Authors working with screening.

**Figure 2:**
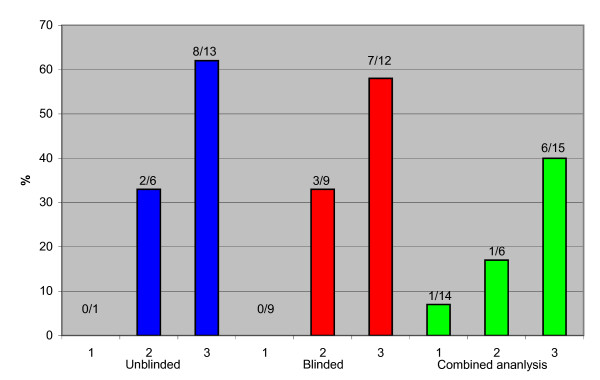
Proportion of the 143 articles eligible for review that downplay or reject overdiagnosis among those that mention it, related to author affiliation. 1: Authors with no apparent conflict of interest. 2: Authors in screening-affiliated specialty or funded by cancer charities. 3: Authorsworking with screening.

Overall, authors working with screening mentioned and acknowledged harms less often than authors not working with screening (29% vs. 40%) but the difference was not statistically significant. Most of the separate 17 items we assessed were mentioned too rarely to draw any conclusions regarding a possible relationship with author affiliation, although there were a few exceptions. We found a trend that authors working with screening were less likely than other authors to acknowledge overdiagnosis and overtreatment (Figure 1) (P = 0.06). We also consistently found that, when they mentioned it, authors working with screening were more likely than other authors to downplay or reject overdiagnosis and overtreatment (Figure 2), and this result was statistically significant in all analyses (P = 0.03). However, the number of events was small. We considered overdiagnosis and overtreatment to be downplayed when described as negligible or non-existent (See Additional file 1 for examples). Other downplayed harms were described as barriers to participation in screening rather than as negative consequences for the women. This was mainly the case when authors described harms such as recalls for further investigation, psychological distress following false positive findings, and pain. These harms could be barriers to participation, but it is not their most important attribute.

Blinding appeared to be effective. In only 16 cases did the blinded observer guess the identity of the authors, or an author's affiliation, and in only one case might this have influenced the judgments of the reported benefits and harms, as the other 15 cases were unambiguous.

## Discussion

We found that scientific articles tend to emphasize the benefits of mammography screening over the harms. The focus on benefits carries a risk of underestimating harms and is in conflict with guidelines on reporting outcomes of medical interventions [1]. Invitations to screening, information material, and newspaper articles have all been demonstrated to present information biased in favour of screening [3-7] As they are all likely to reflect the views presented in the scientific literature, our results could explain a similar focus on benefits in those areas. Failure to cover harms in invitations has serious ethical implications and violates requirements for informed consent [2]. Guidelines on how to develop balanced decision aids exists, and use of such guidelines could help improve the quality of information materials in the future [21,22].

Many authors, in particular those working with screening, downplay or reject even those harms that are arguably the most important: overdiagnosis and overtreatment. This is unfortunate, because we know *a priori*, as stated by the Director of the UK National Screening Committee, that "All screening programmes do harm; some can do good as well" [23]. The most serious harms of screening should therefore have equal priority to the most important benefit – the reduction in breast cancer mortality – if not a higher one, as far more people will experience harm than will benefit [24]. Overdiagnosis and overtreatment were often downplayed as negligible by authors working with screening, but they are not. Assuming a reduction in breast cancer mortality of about 15%, as estimated in the two most recent systematic reviews [24,25]., and 30% overdiagnosis as indicated by the randomised trials [24], screening 2000 women over 10 years would prevent one breast cancer fatality but turn 10 healthy women into cancer patients unnecessarily [24].

Even in comprehensive studies, an evaluation of both benefits and harms of medical interventions is not always performed, or not always published. One recent example is the systematic review on mammography screening performed on behalf of the US Preventive Services Task Force [25]. Although this is the only systematic review performed of all the screening trials after the Cochrane Review of screening was first published in 2001 [26], it only evaluated the reduction in breast cancer mortality. Many of the important harms were mentioned, including overdiagnosis and overtreatment, but none were quantified. The first Cochrane Review is another example. Although the protocol for the review listed use of surgical interventions (biopsy, tumourectomy and mastectomy) and use of chemotherapy and radiotherapy as outcomes to become assessed, the editors of the Cochrane Breast Cancer Group decided to defer presentation and discussion of these results until further editorial review had been completed. These data – that demonstrated substantial overtreatment – were published elsewhere at the time [27] and they are now also available in the updated Cochrane Review [24]. The Cochrane Collaboration is aware of the importance of studying and communicating the harms of interventions, but even with this in mind and with the best of intentions, unpleasant findings can delay publication. The inclusion and quantification of harms such as overdiagnosis and overtreatment in the updated version of the Cochrane Review [24] shows, however, that it is possible to improve reporting and provide a balanced presentation of benefits and harms of interventions. The original review was the first to demonstrate that screening leads to substantial overtreatment, and the estimate of about 30% overdiagnosis and overtreatment has subsequently been supported by large epidemiological studies [28-31]. and with extended data from the Malmö mammography screening trial [32,33]. Hopefully, the increased focus on harms of interventions will be reflected in information material directed towards the public in the future.

Scientific articles on mammography screening favour information on the mortality reduction, and prefer to present this as a relative risk reduction rather than an absolute risk reduction. A relative risk reduction appears more impressive, but tends to make lay people, as well as health professionals, overestimate the obtainable benefit [34]. This problem is known from scientific articles in general, and is particularly important in a screening setting as so few will benefit of the total number screened [35].

We experienced some problems while working on this study. Following a pilot data extraction from 10 articles, we realised that simply recording whether or not a harm was mentioned would provide misleading results. The context in which the harm appeared (framing) had a major influence on the impression the reader would get of its meaning and importance. We therefore decided to evaluate whether or not the harms were downplayed or rejected. This decision was made after the completion of our protocol, but before data extraction began. Our evaluation of whether or not a harm was downplayed could have been influenced by our own views, and others might disagree. We have therefore included a sample of quotes to allow the readers to make their own interpretation (see Additional file 1). However, the focus on benefits in our sample is clear, regardless of this analysis.

As the focus of the screening debate changes, the preferred topics may change as well, and our sample may differ from a sample taken in a different year. However, we find it unlikely that this would play any major role, as the inevitable problem of overdiagnosis with cancer screening has been known for decades.

The results of the blinded data extraction generally supported the findings in the unblinded extraction, but the trends were less pronounced, as could be expected. We thought that the results of our combined evaluation would be somewhere in between the two other results, but this was not the case for the relation between author affiliation and the likelihood for acknowledging overdiagnosis and overtreatment. This is mainly due to a change in affiliation for some authors when we did more extensive searches on the Internet for the combined evaluation.

## Conclusion

Scientific articles tend to emphasize the major benefits of mammography screening over its major harms. This imbalance is related to the authors' affiliation.

## Competing interests

PCG has been involved in a systematic review that questioned the value of screening. AK and KJJ have no conflicts of interest.

## Authors' contributions

PCG conceived the project, participated in data extraction and revised the protocol and manuscript. AK contributed to develop the methods of the review, participated in data extraction and revised the manuscript. KJJ contributed to develop the methods of the review, participated in data extraction and wrote the first draft of the protocol and the manuscript.

## Pre-publication history

The pre-publication history for this paper can be accessed here:



## Supplementary Material

Additional file 1 Quotes and comments Click here for file
