# Supplementary material for: Are benefits and harms in mammography screening given equal attention in scientific articles? A cross-sectional study
Source: BMC Med. 2007 May 30;5:12. doi: 10.1186/1741-7015-5-12 (PMC1896173; doi:10.1186/1741-7015-5-12)
Supplement: Additional file 1 — Quotes and comments [file 1741-7015-5-12-S1.doc]

**Quotes.**

“It seems that overdiagnosis is a relatively minor problem, and one largely confined to DCIS at the prevalent screen.”

**Comment:** *Overdiagnosis is mentioned, but its significance downplayed.*

“Overtreatment can now be markedly reduced and could become negligible in the near future.” **Comment:** *We would consider such a statement as downplaying the importance of overtreatment, the inevitable consequence of overdiagnosis.*

“The trials achieved their results with an acceptably low recall rate of suspicious mammograms, for example 5% to 6% at the first screen and 2,5% to 3% at later screens in the Two-County and Gothenburg Trials.”

**Comment*:*** *The recall rate is characteristically given per screen, not the cumulative risk of being recalled over several rounds, and the importance is downplayed as “acceptably low”.*

“Anxiety before screening or resulting from supplementary imaging work-up, short-term follow-up, cyst aspiration, and biopsy has not dampened the enthusiasm of most women for the value of early detection.”

**Comment:** *Recalls due to false positives are mentioned, but its relevance is only described in terms of its influence on future attendance, not the psychological stress experienced by the attending women.*

“Detection of DCIS is a benefit rather than a risk from screening.”

**Comment:** *It may be to some women, but certainly not to everyone.*

“Women advised to return after screening for supplemental mammographic views may feel anxious for a month or less”

**Comment*:*** *Anxiety caused by false positive result are mentioned, but downplayed, as some women have to go through invasive testing, which could take many months.*

“…our recall rate more closely mirrors the upper values of recall of 17% reported by Gur et al. We, and our patients, are willing to pay the ‘price’ of a slightly higher recall rate and believe, unequivocally, that the benefit of higher recall rate, namely improved carcinoma diagnosis, is well worth the cost”

**Comment:**  *With a recall rate of 17% at each screening round, the number of recalls during 10 rounds would exceed the number of women participating in screening. The European Guidelines indicate 7% as acceptable, and 5% as desirable recall rates at the initial screen, and 5% and 3%, respectively, at subsequent screens. Screened women are not “patients”, and whether women “unequivocally” consider the benefits to outweigh the harms is open to debate. We believe the author downplays the importance of false positive results.*
